# Suppression of Calcineurin Enhances the Toxicity of Cry1Ac to *Helicoverpa armigera*

**DOI:** 10.3389/fmicb.2021.634619

**Published:** 2021-02-11

**Authors:** Jizhen Wei, Xue Yao, Shuo Yang, Shaokai Liu, Shuai Zhou, Junjuan Cen, Xiaoguang Liu, Mengfang Du, Qingbo Tang, Shiheng An

**Affiliations:** ^1^State Key Laboratory of Wheat and Maize Crop Science, College of Plant Protection, Henan Agricultural University, Zhengzhou, China; ^2^Bureau of Agriculture and Rural Affairs of Qixian, Kaifeng, China

**Keywords:** *Helicoverpa armigera*, calcineurin, Cry1Ac, membrane yeast two-hybrid, cell toxicity

## Abstract

Insect resistance to *Bacillus thuringiensis* (Bt) insecticidal proteins has rapidly evolved with the expansion of the planting area of transgenic Bt crops. Pyramiding RNA interference (RNAi) and Bt in crops is urgently needed to counter the rapid increase in pest resistance. The ideal “pyramid” strategy simultaneously targets different action pathways that exert synergetic effects on each other. Here, we identified a dephosphatase, namely, *Helicoverpa armigera* calcineurin (HaCAN), which might enhance the insecticidal activity of Cry1Ac against *Helicoverpa armigera* by regulating immune gene expression via dephosphatase activity, but not by acting as a receptor. Notably, blocking enzyme activity or knocking down endogenous HaCAN significantly promoted the enhancement in Cry1Ac toxicity to insect larvae and cells. Correspondingly, the increase in HaCAN activity reduced the cytotoxicity of Cry1Ac as shown by the heterologous expression of HaCAN. Our results provide a probable that HaCAN is an important candidate gene for pyramiding RNAi and Cry1Ac crops to control cotton bollworm.

## Introduction

Transgenic *Bacillus thuringiensis* (Bt) Cry1Ac crops have provided significantly economic and environmental benefits since they were first introduced in 1996 for the control of major Lepidopteran pests (Mendelsohn et al., 2003; Hutchison et al., 2010; Tabashnik et al., 2010; Naranjo, 2011; Lu et al., 2012; Downes et al., 2017). Therefore, over the past 22 years, Bt crops have been planted by millions of farmers from 1 million in 1996 to over 100 million in 2017 and 2018 (ISAAA, 2018). However, the extensive planting of Bt crops has led to the increase in the selection pressure for Bt resistance, and led to the increasingly rapid evolution of pest resistance. Field-evolved resistance to Bt toxins in some insect pests reduces the benefits of Bt (Tabashnik and Carrière, 2017).

The simultaneous expression of multiple Bt toxins in crops has been considered as a principal strategy for insecticide resistance management to delay pest adaptation (Brévault et al., 2013). However, the durability and efficacy of such Bt crops are reduced by antagonism or cross resistance between Bt toxins (Carrière et al., 2015, 2016). Thus, RNA interference (RNAi) is a promising new alternative for the effective management of insect pests, particularly Bt-resistant insects (Mamta and Rajam, 2017; Ni et al., 2017). RNAi (targeting JHAMT or JHBP) and simultaneous Cry1Ac expression in cotton can be used to control Cry1Ac-resistant cotton bollworm (Ni et al., 2017). However, associated studies have showed that in *Chilo suppressalis* larvae, the knockdown of ATPase subunit A increases resistance to Cry2Aa- and Cry1Ca-expressing rice (Qiu et al., 2019). Therefore, the ideal “pyramid” strategy should simultaneously target different action pathways with synergetic effects on each other. The knockout of an immune gene Sl 102 in *Spodoptera littoralis* was recently verified to improve the effect of spray applications of the Bt-based biopesticide (Xentari^TM^) (Caccia et al., 2020). In fact, feeding with Bt toxins themselves activates a series of immune signal pathways (Chai et al., 2008; Xia et al., 2016; Portugal et al., 2017). Meanwhile, the genes that are involved in the immune pathway can significantly affect insect development (Luo and Dearolf, 2010; Wei et al., 2019a). Thus, immune genes have attracted the attention of researchers, and are considered as candidate genes for the RNAi + Bt for insect control strategy.

Some immune genes, such as *p38*, *jnk*, *C-type lectins*, and *HSP70* that participate in insect defense and immune responses, are reportedly involved in Bt toxicity (Huffman et al., 2004; Chai et al., 2008; Cancino-Rodezno et al., 2010; Oppert et al., 2012; Xia et al., 2016; Portugal et al., 2017). Nevertheless, the roles of these immune genes in combination with Bt toxins in insect control remain elusive. In fact, although many immune-related genes in *Helicoverpa armigera* have been reported (Wang et al., 2010), their functions in Bt toxicity are rarely reported. Our previous study screened one important Ca^2+^-dependent phosphatase, calcineurin (CAN), which serves as an immune activator by binding with relish, a key transcription factor of the immune deficiency (IMD) pathway, to affect the development of cotton bollworm by regulating antimicrobial peptide (AMP) expression (Wei et al., 2019a). CAN has been identified as a dephosphatase of protein phosphatase 2B family and participates in various biological pathways (Furman and Norris, 2014), including immunity pathways (Bueno et al., 2002; Kang et al., 2007; Furman and Norris, 2014; Ballesteros-Martinez et al., 2017). In mammals, CAN is a mediator that participates in innate immunity and anaphylaxis (Bueno et al., 2002; Kang et al., 2007; Ballesteros-Martinez et al., 2017). In insects, Gram-negative bacteria can induce CAN activity to promote the production of relish, and activated relish subsequently induces the expression of antimicrobial genes (Dijkers and O’Farrell, 2007; Li and Dijkers, 2015).

To consider the potential value of the use of CAN in the RNAi + Bt strategy, we investigated CAN role in the insecticidal activity of Cry1Ac against cotton bollworm. In this study, we used the CAN-specific inhibitor, FK506 to block CAN activity, and tested the insecticidal activity of Cry1Ac against cotton bollworm through a diet bioassay. The expression level and enzyme activity of CAN in larvae after Cry1Ac treatment were analyzed. We examined the binding characteristics of HaCAN and Cry1Ac by using the split-ubiquitin based membrane yeast two-hybrid (MYTH) system (Stagljar et al., 1998; Möckli et al., 2007; Snider et al., 2010). We also conducted *in vitro* gain and loss of function analyses by expressing endogenous *HaCAN* and *HaCAN* double-stranded RNA (dsRNA) in Sf9 cells and Helicoverpa *zea* midgut cells, respectively. Our findings indicated that *HaCAN* was not a receptor of Cry1Ac, but was rather an activator of the immune gene. *HaCAN* was involved in the insecticidal activity of Cry1Ac against cotton bollworm. Our results provided a probable that *HaCAN* is an important candidate gene for pyramiding RNAi and Cry1Ac in crops to control cotton bollworm.

## Materials and Methods

### Insects and Cell Lines

*Helicoverpa armigera* larvae were purchased on October 2018 (Henan Jiyuan Baiyun Industry Co., Ltd.) and maintained in the laboratory condition with artificial diet (Zhao et al., 2009).

*Helicoverpa zea* midgut cell line RP-HzGUT-AW1 (MG) was generously provided by Dr. Xianchun Li (University of Arizona, Tucson, AZ, United States) (Goodman et al., 2004). *Spodoptera frugiperda* cell line (Sf9) and MG cell line were routinely maintained at a constant temperature incubator, and the culture mediums were prepared as the description in Wei et al. (2016, 2018a, 2019b).

### Bt Toxins

Cry1Ac protoxin and activated Cry1Ac were purchased from Beijing General Pest Biotech Research Co., Ltd.^1^. The activated Cry1Ac proteins were re-dissolved in Excell 420 insect serum-free medium or Sf-900 II SFM media following the previous description (Wei et al., 2016).

### Bioassays

We tested the susceptibility of second instar larvae to Cry1Ac (2 μg/mL, about the 40% mortality for second instar larvae) toxins or FK506 (APEBIO; 10, 20, and 50 μM) singly or in Cry1Ac (2 μg/mL) + FK506 (10, 20, and 50 μM) combinations. Before the bioassay, the newly molting second instar larvae were first starved for 12 h before diet bioassay. The 50 mM pH 10.0 Na_2_CO_3_ (dissolve Cry1Ac, as a buffer control to adjust the mortality of Cry1Ac treatment), DMSO (dissolve FK506, as a buffer control to adjust the mortality of FK506 treatments), and Na_2_CO_3_ + FK506 (as a buffer control to adjust the mortality of Cry1Ac + FK506 treatments) treatments were used as buffer controls, and the same volumes of buffer as that of the treatments were used. All these toxins or inhibitors were mixed with artificial diet. For each treatment (single toxin, combination, or control), 48–64 same size second instar larvae (16 larvae for each replicate) were transferred to the wells (one larva per well) in 24-well plates. The larvae mortalities daily until 7 days’ post-exposure to these above treatments were recorded.

### The Analysis of the Expression and Activity of CAN Induced by Cry1Ac

To investigate the effect of feeding with 25 μg/mL Cry1Ac (about the 30% mortality for fifth instar larvae) on *HaCAN* expression, 10 larvae (fifth instar) were treated with three biological replicates. After feeding at different time (6, 12, 24, 48, and 72 h), the larvae were collected and corresponding midgut tissues were harvested.

To analyze the effect of feeding with Cry1Ac, FK506, and Cry1Ac + FK506 on CAN activity, the new molting of fifth instar larvae was selected. 12 h after starvation, the larvae were refed with the artificial diet containing 25 μg/mL Cry1Ac (about the 30% mortality for fifth instar larvae), 50 μM FK506, and Cry1Ac (25 μg/mL) + FK506 (50 μM) for 72 h, respectively. The 50 mM pH 10.0 Na_2_CO_3_ (dissolve Cry1Ac) and DMSO (dissolve FK506) were used as buffer controls. The larvae were collected and the midgut tissues were harvested.

### Membrane Yeast Two-Hybrid (MYTH) Screen Assay

A full open reading frame (ORF) of Cry1Ac was PCR-amplified from Bt *HD73* plasmids, using the gene-specific primers pBT3-N-Cry1Ac (forward: ATCGAATTCCTGCAG *GGCC*ATTACGGCCATGGATAACAATCCGAACATC; reverse: TACTTACCATGG*GGCC*GAGGCGGCCCTATTCCTCCATAAG GAGTAATTCC; with the *Sfi*I restriction site). After the *Sfi*I digestion of the PCR products, *Cry1Ac* was cloned into the bait vector pBT3-N (DUALsystems BioTech). *HaCAN* was cloned into expression vector pPR3-N (Figure 4B), the construction of *HaCAN* was prepared in our previous study, and it had been shown that pPR3-N-CAN can express in yeast cells and work well (Wei et al., 2019a).

Four treatments were introduced into competent cells of the Yeast strain NMY51 (DUAL systems BioTech) with lithium acetate (LiAc)/PEG-mediated transformation, including 100 ng pTSU2-APP and 100 ng pNubG-Fe65, 100 ng pBT3-N-Cry1Ac and 100 ng pOst1-NubI, 100 ng pBT3-N-Cry1Ac and 100 ng pPR3-N, and 100 ng pBT3-N-Cry1Ac and 100 ng pPR3-N-HaCAN. The detail of co-transformation method followed the previous description (Wei et al., 2019a). Then, the interaction pairs were screened on four SD (Synthetic Defined Drop-out Medium) plates as the previous description (Wei et al., 2019a). Finally, we photographed the colonies of each prey and bait construct pair on the above four plates.

### Plasmid of *HaCAN*-GFP-pIEx Constructs

The whole ORF was cloned into pIEx-RFP vector [kindly provided by Professor Xiaofan Zhao (Shandong University, School of Life Sciences)]. First, we designed gene-specific primers of *HaCAN* in Supplementary Table S1. *HaCAN* ORF was amplified with the primers *HaCAN*-5′ Sac I and *HaCAN*-5′ Bgl II and template *HaCAN*-pMD-18T DNA (Wei et al., 2019a). PCR products were cloned into pGEM^®^-T vector (Promega, Madison, WI, United States), followed by restriction enzyme digestion with Sac I and Bgl II (New England Biolabs, Ipswich, MA, United States). The pIEx-RFP vector was also digested with Sac I and Bgl II at 37°C overnight. Ligation reaction of pIEx-RFP with *HaCAN* was carried out at 16°C overnight and was transfected to DH5α cells. Recombinant plasmids were extracted for subsequent use.

### Double-Strand RNA Synthesis

*HaCAN* and *EGFP* dsRNA were synthesized using the MEGAscript RNAi kit (Ambion, Vilnius, Lithuania) as the description of using gene-specific primers containing T7 polymerase sites (all the related primers are listed in Supplementary Table S1) to prepare the templates for dsRNA synthesis (Du et al., 2012, 2017). A 466 bp cDNA fragment of *HaCAN* was PCR-amplified from HaCAN-pMD-18T (Wei et al., 2019a) to generate the template for *in vitro* dsRNA synthesis. Amino acid sequence analysis indicated that *CAN* in *H. armigera* shared 99% amino acid identity with *H. zea* CAN homolog (unpublished data). The same fragment of *HaCAN* and *HzCAN* can be used in *H. zea* MG cells to test the function of CAN. The enhanced green fluorescent protein (EGFP) template (a negative control) was prepared as the previous descriptions in Du et al. (2012). The synthesized dsRNAs of *HaCAN* and *EGFP* were then eluted in DEPC water and dsRNA concentrations were measured by a BioPhotometer (Eppendorf, Hauppauge, NY, United States) (Du et al., 2017).

### Transient Transfection and Cell Bioassay

Sf9 and *H. zea* midgut cells were seeded onto a 12-well plate (costar, 9 × 10^5^cells/well), allowing cells to attach for 3 h. Then, cells were transiently (5 h) transfected with 1.5 μg/well pIEx-RFP-*CAN* plasmid (or 50 mM DsCAN) using FuGENE HD Tran section Reagent (Promega; 8 μL per well). Empty pIEx-RFP (or DsEGFP) vector was used as control. Five hours after transfection, transfection mixture was removed and replaced by the newly supplemented medium (according to the description in Wei et al., 2016, 2018a, 2019b). Sixty-four hours after cell maintenance at 28°C, the cells that transfected pIEx-RFP-*CAN* or pIEx-RFP were observed under the fluorescence microscope (Zeiss, LSM780) to check the expression of proteins. After 64 h post-transfection, the cells were also collected and the concentration of cells was measured with hemocytometer by using trypan blue; then the treated cells were reseeded in a 96-well micro-plate (costar) for 100 μL of cells (10,000 cells), allowing cells to attach about 2 h. We tested the cytotoxicity of the treated cells by using Cry1Ac toxin (100 μg/mL for expressing *HaCAN* and 50 μg/mL for knockdown of *HaCAN*) for 4 h; finally, the cell mortality was calculated under the inverted microscope (OPTIKA IM-5) (Wei et al., 2016, 2018a, 2019b). The remaining treated cells of each independent transfection were collected, centrifuged at 1000 × *g* for 10 min, washed with an equal volume of cold PBS buffer for three times, and then stored them at −80°C for subsequent RNA or protein extraction to make sure the successful transfection.

### Protein Extraction and Western Blot

The cell proteins were extracted using 100 μL RIPA lysis buffer (Wei et al., 2016) with 10 μL PMSF (to prevent the degradation of proteins) and 25 μL 5× SDS loading buffer (Wei et al., 2016); 20 μL of protein mixture was separated on 10% sodium dodecyl sulfate polyacrylamide gel electrophoresis (SDS-PAGE) and electroblotted to 0.45 μm polyvinylidene difluoride (PVDF) (Solarbio) membrane in 25 mM Tris, 192 mM glycine, 10% methanol, PH 8.3. The membrane was blocked with PBST (137 mM NaCl, 2.7 mM KCl, 10 mM Na_2_HPO_4_, 2 mM KH_2_PO_4_, pH 7.4, 0.1% Tween 20) and 5% fat-free milk (Sangon, Shanghai) for 1 h at 25°C. Then mouse-anti-RFP (1:1000) was added to the blocking buffer; 1 h after incubation at 25°C, the membrane was washed for five times (5 min for each time) using PBST. Then sheep anti-mouse secondary antibody (Beijing ComWin Biotech Co., Ltd.) (1:10,000) was added to PBST for 1 h at 25°C with constant shaking. Finally, immune-band was detected with immobilon western electrochemiluminescence HRP substrate (ECL, Abbkine) and with Tanon-4600 photographic.

### Determination of CAN Activity

The dephosphorylase activity of CAN was tested using a Calcineurin Activity Assay Kit [cat. no. ab139461; Abcam (Cambridge, MA, United States)] according to the previously described methods (Li et al., 2017; Zhao et al., 2018).

### RT-PCR

Total RNA was extracted from collected cells and midgut samples with Total RNA Isolation Mini kit (Ambion, Vilnius, Lithuania). The resultant RNA samples were treated with DNase I (Fermentas, EU) to remove potential DNA contamination. RNA samples were reverse transcribed into first-strand cDNA with First-Strand cDNA Synthesis Kit (Fermentas, EU).

To test the expression levels of *HaCAN*, qRT-PCR was carried out using Applied Biosystems 7500 Fast Real-Time PCR system (ABI, Carlsbad, CA, United States). *H. armigera* ribosomal protein gene 18S (*RP18S*) and *EF1-*α were used as reference genes (Du et al., 2017; Wei et al., 2019a). qRT-PCR reaction system, thermocycler conditions, melting curve analysis, and the calculation method for expression level of the *HaCAN* followed previously described methods (Liu et al., 2015; Wei et al., 2019a). The primers used for the RT-qPCR in the methods are listed in Supplementary Table S1; the sizes of amplicons, amplification efficiency, and annealing temperature of each pair of primers are also listed in Supplementary Table S1.

### Statistical Analysis

We compared the expected mortality for each combination of FK506 and Cry1Ac with the observed mortalities of each combination to evaluate the interactions between FK506 and Cry1Ac (Tabashnik et al., 2013; Wei et al., 2015). The expected mortality of each combination is calculated using the following formula:

(1-[S×(Cry1Ac)OBSS](FK506)OBS)×100%(Tabashnik et al., 2013;Wei et al.,)

where S_(Cry__1__*Ac)OBS*_ is the corrected observed survival rate under the treatment of Cry1Ac and S_(FK__506__)OBS_ is the corrected observed survival rate under the treatment of FK506. The differences between expected and observed mortalities of each combination were analyzed by Student’s *t*-test (DPSSOFT: DPS7.05).

Significant difference in the expression level of *HaCAN* between CK (buffer control) and treatments (feeding Cry1Ac) at each different time point was compared with Student’s *t*-test (DPSSOFT: DPS7.05).

Significant difference in the enzyme activities of HaCAN at different treatments was compared with the Tukey’s test with *P* < 0.05 (DPSSOFT: DPS7.05).

Cell mortality was calculated with the Abbott formula (Abbott, 1925) as the description in Wei et al. (2016, 2019b). Significant differences in cell mortalities, relative expression level, and enzyme activities of HaCAN between CAN-GFP-pIEx (dsCAN) and GFP-pIEx (dsEGFP) were compared using Student’s *t*-test (DPSSOFT: DPS7.05).

## Results

### *HaCAN* Expression Was Induced by Cry1Ac

**FIGURE 1:**
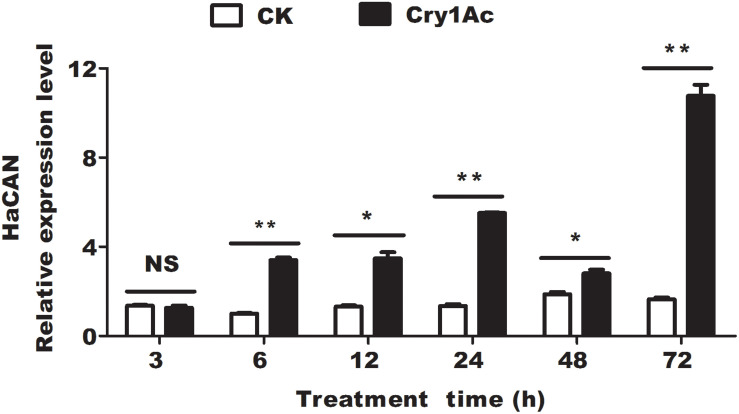
The effect of feeding with CAN inhibitor FK506 on the expression level of HaCAN. Each error bar represents the standard error of the mean from three biological replicates. Significant differences between CK (buffer control) and treatments (feeding Cry1Ac) at different time points are indicated with asterisks (*P < 0.05, based on Student’s *t*-tests, DPS7.05).

We investigated the expression levels of *HaCAN* transcript at 6, 12, 24, 48, and 72 h after feeding with Cry1Ac. Comparison with the corresponding controls revealed that *HaCAN* transcript had significantly increased (6 h, *P* = 0.0001; 12 h, *P* = 0.0018; 24 h, *P* = 0.0001; 48 h, *P* = 0.0099; 72 h, *P* = 0.0001) (Figure 1). Notably, a 6.53-fold increase in the expression level of *HaCAN* transcript was observed at 72 h after Cry1Ac treatment (Figure 1).

### HaCAN Activity Was Induced by Cry1Ac and Inhibited by FK506

**FIGURE 2:**
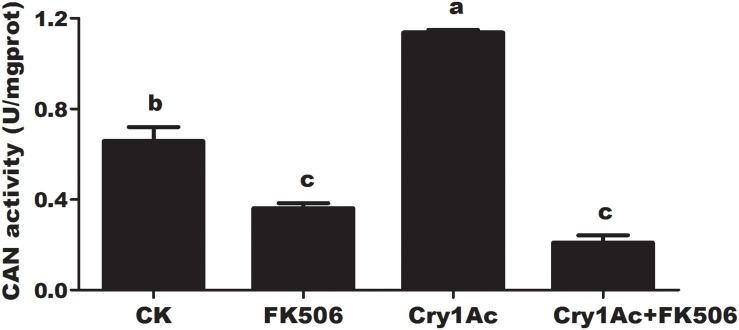
The effect of feeding with FK506, Cry1Ac, and Cry1Ac + FK506 on HaCAN activity in the midgut of larvae. Each error bar represents the standard error of the mean from three biological replicates. Significant differences among the different treatments are indicated with different lowercase letters (*P* < 0.05, based on Tukey’s test, DPS7.05).

FK506 feeding caused the significant suppression of HaCAN activity by comparing with the buffer control at 72 h. Feeding with 25 μg/mL Cry1Ac led to a significant increase in HaCAN activity. However, the increase in HaCAN activity induced by Cry1Ac was attenuated by FK506 (*P* = 0.0001; Figure 2).

### Inhibition of HaCAN Activity Increased the Insecticidal Activity of Cry1Ac to Larvae

**FIGURE 3:**
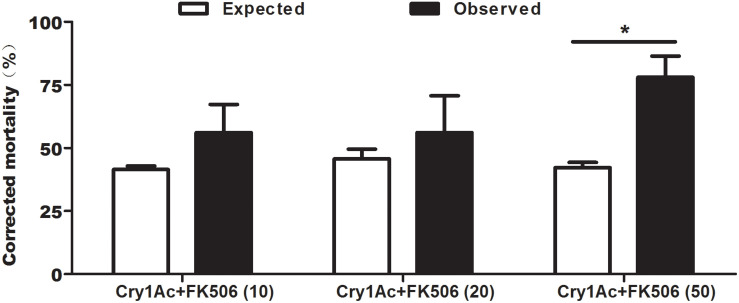
Expected versus observed mortalities caused by the combinations of Cry1Ac and FK506 against *H. armigera*. Each error bar represents the standard error of the mean from three biological replicates. 10, 20, and 50 indicate the concentrations of FK506 in μM. Asterisk shows significant differences between observed and expected mortality at *P* < 0.05 level (based on Student’s *t*-tests, DPS7.05).

To investigate the effect of FK506 on Cry1Ac toxicity, FK506 was added to an artificial diet containing Cry1Ac. Feeding with 10 μM FK506 + Cry1Ac and 20 μM FK506 + Cry1Ac resulted in considerably higher observed corrected mortalities than expected corrected mortalities (Figure 3). When the larvae were fed on artificial diets containing a high dose of FK506 (50 μM FK506 + Cry1Ac), the observed corrected mortality reached 78.05%, whereas the expected corrected mortality was only 42.18% (Figure 3). The mixture of 50 μM FK506 + Cry1Ac significantly improved the insecticidal activity of Cry1Ac against cotton bollworm (*P* = 0.0473) (Figure 3).

**FIGURE 4:**
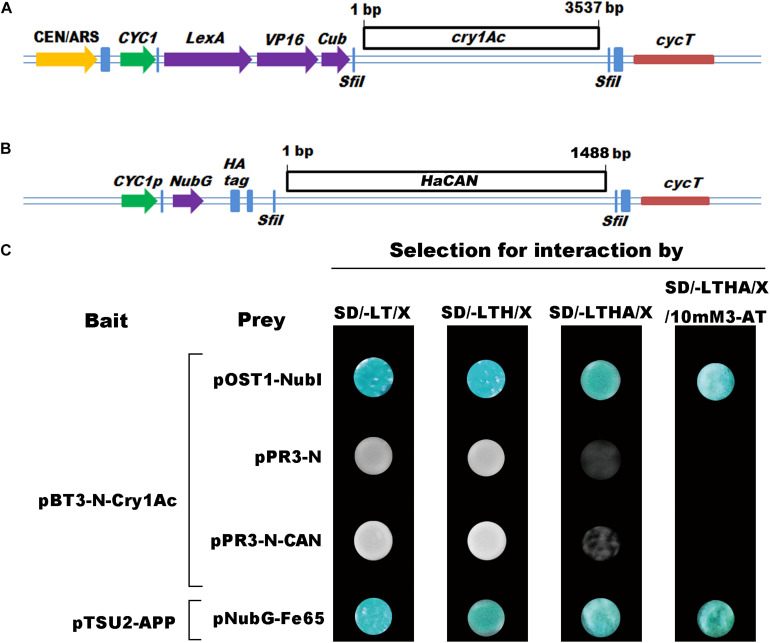
The interactions between Cry1Ac and HaCAN based on yeast two-hybrid system. Diagrammatic representation of the bait **(A)** and prey **(B)** constructs. SD/-LT/X (SD-leucine-tryptophan + 40 mg/L X-Gal media) (X-Gal soluted in N,N-dimethylformamide), SD/-LTH/X (SD-leucine-tryptophan-histidine + 40 mg/L X-Gal media), SD/-LTHA/X (SD-leucine-tryptophan-histidine-adenine + 40 mg/L X-Gal media), and SD/-LTHA/X/10mM3-AT (SD-leucine-tryptophan-histidine-adenine + 40 mg/L X-Gal media + 10 mM 3-AT) plates, respectively. **(C)** Representative growth of NMY51 yeast cells co-transformed with one of the seven pairs of prey and bait constructs on four SD medium plates with increasing selection stringency. The treatment of pTSU2-APP and pNubG-Fe65 was positive control. The treatment of pBT3-N-Cry1Ac and pPR3-N was negative control.

### Interaction Between HaCAN and Cry1Ac

Cry1Ac was inserted into the pBT3-N bait vector, which was fused with the C-terminal half of ubiquitin (Cub) and an artificial transcription factor (LexA-VP6) (Figure 4A). Before the interactions, the positive control of the transformants of pTSU2-APP and pNubG-Fe65 was found to work effectively (Figure 4C; Yu et al., 2015). This result verified the utility of the split-ubiquitin-based MYTH system to identify protein–protein interactions, including cytosolic proteins. The transformants of pBT3-N-Cry1Ac and pOst1-NubI grew well and turned blue in β-galactosidase assay on SD/-LT/X (X-α-Gal dissolved in N, N-dimethylformamide), SD/-LTH/X, SD/-LTHA/X, and SD/-LTHA/X/10mM3-AT plates (Figure 4C). These results showed that plasmids of pBT3-N-Cry1Ac worked well in this experiment. The transformations of pBT3-N-Cry1Ac and pPR3-N did not turn blue in the β-galactosidase assay of SD/-LT/X, SD/-LTH/X, SD/-LTHA/X, and SD/-LTHA/X/10mM3-AT plates (Figure 4C), which indicated that Cry1Ac could not interact with the NubG fused non-sense peptide. In our previous study, we prepared a *HaCAN* construct (Figure 4B) and showed that pPR3-N-CAN can be expressed in yeast cells and work well (Wei et al., 2019a). We had verified the interaction between Cry1Ac and HaABCC2, a known receptor of Cry1Ac by co-transforming the Cry1Ac bait construct pBT3-N-Cry1Ac and the HaABCC2 prey construct pPR3-N-HaABCC2 (our submitted paper), which indicated that this split-ubiquitin MYTH system could be used to identify the interaction between Bt toxins and receptors. The transformants of pBT3-N-Cry1Ac and pPR3-N-HaCAN did not turn blue in β-galactosidase assay of SD/-LT/X, SD/-LTH/X, SD/-LTHA/X, and SD/-LTHA/X/10 mM 3-AT plates. This result indicated that Cry1Ac and HaCAN could not interact directly (Figure 4C).

### Heterologous Expression of HaCAN Affected Cry1Ac Cytotoxicity Against Sf9 Cells

**FIGURE 5:**
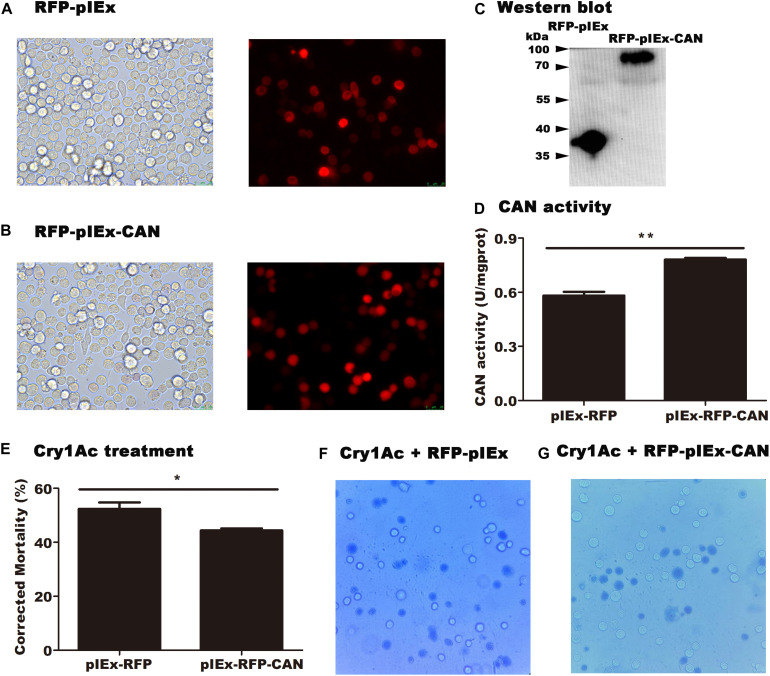
Impact of the heterologously expressed *HaCAN* on the cytotoxicity of Cry1Ac to midgut cells. **(A)** The Sf9 cells that transfected GFP-pIEx (empty vector). **(B)** The Sf9 cells that transfected GFP-pIEx-CAN. **(C)** Western blot analysis of HaCAN expression in the transfected cells. **(D)** The determination of HaCAN activity. **(E)** Cell mortalities caused by 100 μg/mL activated Cry1Ac. **(F,G)** Photographs are representative of 400x views of the two treatments under an inverted microscope (OPTIKA IM-5). The blue cells represent dead cells, which were stained blue by trypan blue. Each error bar represents the standard error of the mean from three transfection replicates. Asterisks show significant differences in expression levels, enzyme activities, and mortalities between each treatment (*P* < 0.05, Student’s *t*-test, DPSSOFT: DPS7.05).

pIEx-RFP-CAN and pIEx-RFP (empty vector) plasmids were transfected into Sf9 cells. Red fluorescence was observed under a fluorescence microscope, indicating that the corresponding protein had been successfully expressed in the cells (Figures 5A,B). After the successfully expression of *HaCAN* in Sf9 cells, the HaCAN proteins were detected in transfected cells as shown by Western blot results (Figure 5C). Correspondingly, HaCAN activity was also significantly improved (Figure 5D), compared with that in the control (*P* = 0.001) (Figure 5D). Importantly, the overexpression of HaCAN led to a significant reduction in cell susceptibility to Cry1Ac (Figure 5E) (*P* = 0.0347). Photographs also showed that the overexpression of HaCAN in Sf9 cells can could reduce the cytotoxicity of Cry1Ac (Figures 5F,G).

### Suppressing HaCAN Increased the Susceptibility of Midgut Cells to Cry1Ac

**FIGURE 6:**
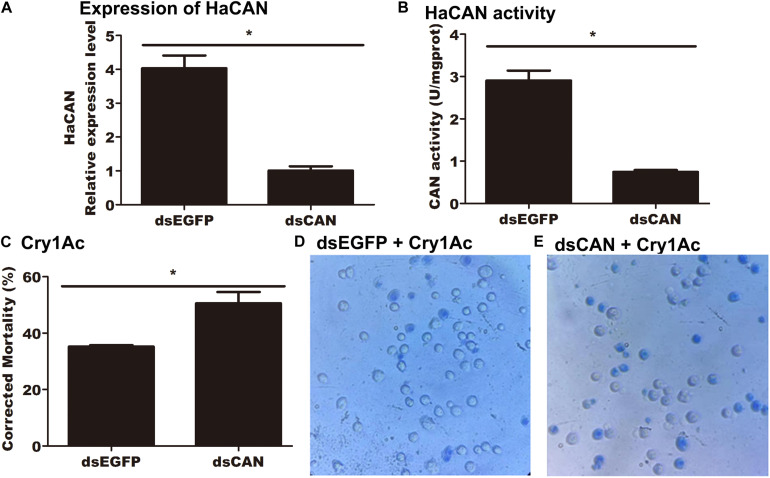
Impact of silencing *HaCAN* on the cytotoxicity of Cry1Ac to midgut cells. **(A)** Expression levels of *HaCAN* in the midgut cells. **(B)** The determination of HaCAN activity. **(C)** Cell mortalities caused by 50 μg/mL activated Cry1Ac. **(D,E)** Photographs are representative of 400x views of the two treatments of the cell lines under an inverted microscope (OPTIKA IM-5). The dead cells were stained blue by trypan blue. Each error bar represents the standard error of the mean from three transfection replicates. Asterisks show significant differences in expression levels, enzyme activities, and mortalities between each treatment (*P* < 0.05, Student’s *t*-test, DPSSOFT: DPS7.05).

qRT-PCR detected a significant reduction in the mRNA level of *HaCAN* in midgut cells transfected with 50 nM *HaCAN* dsRNA (Figure 6A) compared with that in the control transfected with 50 nM EGFP dsRNA control (*P* = 0.0017) (Figure 6A).

HaCAN activity was measured after the successful knockdown of *HaCAN* by RNAi in midgut cells. The result demonstrated that HaCAN activity in the treated cells was significantly reduced in comparison with that in the control cells (*P* = 0.0008) (Figure 6B).

*HaCAN* dsRNA-transfected midgut cells were further treated with 50 μg/mL of activated Cry1Ac. The results demonstrated that the cells transfected with *HaCAN* dsRNA showed significantly higher mortality than those transfected with EGFP dsRNA (increased by 15.39%) (*P* = 0.0349) (Figure 6C). Photographs also showed that the decrease in HaCAN mRNA in midgut cells resulted in the increase in the cytotoxicity of Cry1Ac to cells (Figures 6D,E).

## Discussion

To study the mode of action of Bt, more attention was paid to explore the functional binding receptors with Bt toxin. To date, four main types of receptors, including cadherin, aminopeptidase, alkaline phosphatase, and ATP-binding cassette (ABC) transporters, have been identified (Wei et al., 2019c) on the basis of their capabilities to bind with Bt proteins and their involvement in Bt resistance. However, differentially expressed genes in biochemical pathways, including the hydrolase, digestive, catalytic, immune, and detoxification pathways, may be enriched by genomic or proteomic response or resistance to Bt (Chen et al., 2010; Yuan et al., 2011; Lei et al., 2014; Nanoth Vellichirammal et al., 2015; Xia et al., 2016; Zhang et al., 2017; Wei et al., 2018b). Importantly, these differentially expressed genes such as heat shock proteins (HSPs) and V-ATPase subunits, also participate in the toxicity of Bt to insects (Yuan et al., 2011; Xia et al., 2016; Zhang et al., 2017; Rubio-Infante et al., 2018). In *Plutella xylostella*, Hsp90 can bind with Cry1A proteins to form a positive loop to protect the Cry protoxin from gut protease degradation (García-Gómez et al., 2019). In the present study, HaCAN was significantly upregulated by Cry1Ac (Figure 1). Investigations on the Cry1Ac-induced genes may help elucidate the mode of action of Bt and avoid the development of resistance.

In addition, this study also confirmed that protein phosphorylation process might participate in Bt toxicity. As reported, the phosphorylated activation of MAPK p38, which acts as a defense response, is involved in the initial response to CF1 cells after intoxication with Cry1A toxins (Portugal et al., 2017). Furthermore, the Cry1Ac protoxin can bind with HSP70 on the cell surface, and thereby participate in Cry1Ac protoxin-induced macrophage activation to reduce Cry1Ac protoxin-induced ERK1 phosphorylation (García-Gómez et al., 2019). A further in-depth study on *P. xylostella* demonstrated that the altered protein phosphorylation levels of p38, JNK, and ERK in the MAPK signaling cascade may possibly trigger the differential expression of aminopeptidase N and other midgut receptors, thereby leading to insect resistance to Cry1Ac (Guo et al., 2020). These studies hinted that protein kinase in signal transduction is involved in Bt toxicity. In the present study, we found for the first time that a dephosphorylase, HaCAN, affected Cry1Ac insecticide activity (Figures 2, 3, 5, 6).

The findings reported in this paper showed that although HaCAN affected the insecticidal activity of Cry1Ac against cotton bollworm (Figures 3, 5, 6), HaCAN was not a target receptor of Cry1Ac (Figure 4). However, how HaCAN defends the insect against Bt toxin damage remains a question? In yeast and mammal, CAN activates the transcription factor nuclear factor of activated T cells (NFAT) *via* dephosphorylation, therefore regulating the expression of AMPs and other immune-related genes through the Toll or IMD pathways (Aramburu et al., 2000). In *Drosophila*, CAN activity can be induced by Gram-negative bacteria (Dijkers and O’Farrell, 2007; Li and Dijkers, 2015), leading to relish activation and finally promoting antimicrobial gene expression through the IMD or Toll pathway (Meng et al., 1999; Dijkers and O’Farrell, 2007; Xu et al., 2012; Li and Dijkers, 2015; Yang et al., 2015). Our previous findings indicated that CAN can bind with relish to regulate the expressions of AMPs (attacin, cecropin D, and gloverin) when the insect injected with Gram-negative bacteria in *H. armigera* (Wei et al., 2019a). However, a study confirmed that AMPs produced by Trichoplusia *ni* in response to Bt toxins are not Gram-specific (Tamez-Guerra et al., 2008). Importantly, in insect detoxification reactions to Bt, AMPs are induced in response to Bt challenge and also affect the insecticidal activity of Bt to insect (Ma et al., 2005a,b; Hwang and Kim, 2011). The knockdown of gloverin, an AMP, significantly increases the toxicity of Bt to Spodoptera *exigua* larvae (Hwang and Kim, 2011). Bt-resistant *Galleria mellonella* has a more intact midgut than susceptible insects because it secretes antimicrobial factors (such as gloverin and cecropin) into the gut lumen for the external mitigation of Bt (Dubovskiy et al., 2016). Just as it reported, one of the insect defense mechanisms against Bt toxins is the activation of the cellular detoxification reaction, which reduces the amount of Bt endotoxin within the lumen (Rahman et al., 2004, 2006; Ma et al., 2005a,b; Hernández-Martínez et al., 2010). Therefore, we speculated that HaCAN might regulate the expression of AMPs in the insect against Bt toxin damage. However, a more detailed mechanism underlying HaCAN and AMPs defense against Cry1Ac needs to be studied in the future.

But what is important that HaCAN acts as an important gene in the development of cotton bollworm (Wei et al., 2019a). The data obtained in the present study indicated that “pyramids” of RNAi (targeting CAN) and Cry1Ac crop might work well to control cotton bollworm because the low expression or enzyme activity of HaCAN could increase the insecticidal activity of Cry1Ac (Figures 2, 3, 5, 6). In addition, this transgenic strategy might be an ideal combination, because the HaCAN-mediated dephosphorylation of acetyl-coA carboxylase regulates pheromone biosynthesis activating neuropeptide-induced sex pheromone biosynthesis (Du et al., 2017) and mating behavior in adults (Zhao et al., 2018). Considering the important roles of *HaCAN* in the larvae and adults, it provided a good clue that CAN might be an excellent candidate gene for use in “pyramids” strategy by the simultaneous expression of RNAi (targeting CAN) and Cry1Ac in crop to control cotton bollworm. However, the detailed effects of RNAi (targeting CAN) and Cry1Ac in larvae and adults should be evaluated in the future.

## Data Availability Statement

The raw data supporting the conclusions of this article will be made available by the authors, without undue reservation.

## Author Contributions

JW, QT, MD, and SA conceived and designed the experiments and wrote the manuscript. XY, SY, SL, JC, XL, and JW conducted the experiments and analyzed the data. All authors read and approved the manuscript.

## Conflict of Interest

The authors declare that the research was conducted in the absence of any commercial or financial relationships that could be construed as a potential conflict of interest.

## Footnotes

1 www.genralpest.com
