# Supplementary material for: Suppression of Calcineurin Enhances the Toxicity of Cry1Ac to Helicoverpa armigera
Source: Front Microbiol. 2021 Feb 11;12:634619. doi: 10.3389/fmicb.2021.634619 (PMC7904703; doi:10.3389/fmicb.2021.634619)
Supplement: Supplementary file 1 [file Table_1.docx]

**Suppression of calcineurin enhances the toxicity of Cry1Ac to *Helicoverpa armigera***

Jizhen Wei^1^, Xue Yao^1^, Shuo Yang^1^, Shaokai Liu^1^, Shuai Zhou^1^, Junjuan Cen^2^, Xiaoguang Liu^1^, Mengfang Du^1*^, Qingbo Tang^1*^, Shiheng An^1*^

^1^State key Laboratory of Wheat and Maize Crop Science/College of Plant Protection, Henan Agricultural University, Zhengzhou, 450002, China.

^2^Bureau of Agriculture and Rural Affairs of Qixian, Kaifeng, 475200, China.

*Author to whom correspondence should be addressed; Mengfang Du, E-mail: dumengfang@163.com; Qingbo Tang, E-mail: qingbotang@126.com; Shiheng An, E-mail: [anshiheng@aliyun.com](mailto:anshiheng@aliyun.com)

Table S1 The primers used in this study.

| Primer name | Applications | Sequence(5'→3') | Sizes of amplicons (bp) | Amplification efficiency (%) | Annealing temperature (°C) |
| --- | --- | --- | --- | --- | --- |
| HaCAN-RT-F | Quantitative Real-time PCR | GTCGCATGTAAACAGCAACG | 171 | 99 | 58 |
| HaCAN-RT-R |  | CGACAATTCCCTTCAAATCC |  |  |  |
| Ha18S-RT-F |  | GCATCTTTCAAATGTCTGC | 230 | 97 | 58 |
| Ha18S-RT-R |  | TACTCATTCCGATTACGAG |  |  |  |
| EF1a-RT-F |  | GCCTGGTACCATTGTCGTCT | 153 | 100 | 58 |
| EF1a-RT-R |  | GTAACCACGACGCAACTCCT |  |  |  |
| HaCAN-ds-F | Amplification for fragment of dsRNA | TGTCTTTACCTGGTCCCT | / | / | / |
| HaCAN-ds-R |  | AATGTGCGTTGCTGTTT |  |  |  |
| HaCAN-T7-F |  | GATCACTAATACGACTCACTATAGGGAGATGTCTTTACCTGGTCCCT | / | / | / |
| HaCAN-T7-R |  | GATCACTAATACGACTCACTATAGGGAGAAATGTGCGTTGCTGTTT |  |  |  |
| HaEGFP-ds-F |  | CCTGAAGTTCATCTGCACCAC | / | / | / |
| HaEGFP-ds-R |  | CTCCAGCAGGACCATGTGATC |  |  |  |
| HaEGFP-T7-F |  | GATCACTAATACGACTCACTATAGGGAGACCTGAAGTTCATCTGCACCAC | / | / | / |
| HaEGFP-T7-R |  | GATCACTAATACGACTCACTATAGGGAGACTCCAGCAGGACCATGTGATC |  |  |  |
| HaCaN-pIEX-F | Vector construction | CGTTAACACGTCAAGAGCTCATGTCCGGGAGCAATG | / | / | / |
| HaCaN-pIEX -R |  | CTGCAGGCGCGCCGAGATCTGCGAATGAGCATTGC |  |  |  |
